# Biomarkers of metal toxicity in embryos in the general population

**DOI:** 10.1002/jcla.22974

**Published:** 2019-07-11

**Authors:** Ou Jie, Ping Peng, Lin Qiu, Lirong Teng, Chunying Li, Jianhua Han, Xinyan Liu

**Affiliations:** ^1^ Department of Obstetrics and Gynecology, Chinese Academy of Medical Sciences Peking Union Medical College Hospital, Peking Union Medical College Beijing China; ^2^ Department of Clinical Laboratory, Chinese Academy of Medical Sciences Peking Union Medical College Hospital, Peking Union Medical College Beijing China

**Keywords:** biomarker, embryotoxicity, public exposure, reproductive effect, spontaneous abortion, Toxic metal

## Abstract

**Background:**

With the development of industrialization, public exposure to toxic metals could occur everywhere, eventually affecting individuals’ reproductive systems and even embryos and leading to early pregnancy loss. The aim of the study was to determine the profile of toxic metal levels in pregnant women in the general population and to identify biomarkers for metal toxicity in embryos.

**Methods:**

A case‐control study with pregnant women was conducted at Peking Union Medical College Hospital in 2016‐2018. Women who experienced spontaneous abortion within 12 weeks of gestation comprised the case group, and women with pregnancies showing fetal cardiac activity who requested an induced abortion almost simultaneously were included in the control group. Blood and urine specimen were tested for concentrations of cadmium, chromium, selenium, arsenic, and mercury.

**Results:**

A total of 195 patients were enrolled, with 95 in the case group and 100 in the control group. Significant differences in gravidity, parity, history of miscarriage, mean blood cadmium levels, and mean urine chromium levels were present between the two groups (P1 = 0.013, P2 = 0.000, P3 = 0.000, P4 = 0.002, P5 = 0.046); the odds ratios in the spontaneous abortion with blood cadmium >0.4 µg/L, urine chromium >2 µg/L, gravity <3, parity <2, and history of miscarriage >1 compared with the induced abortion group were 1.26 (1.09, 1.85), 1.56 (1.23, 2.53), 1.39 (1.17, 1.98), 1.72 (1.21, 4.62), and 1.18 (1.06, 1.65), with *P*‐values of 0.003, 0.031, 0.003, 0.247, and 0.001, respectively.

**Conclusion:**

Blood cadmium and urine chromium levels are two possible biomarkers of toxic metal embryotoxicity in the general population, which means that in the general population, blood cadmium >0.4 µg/L or urine chromium >2 µg/L might indicate an increased risk of spontaneous abortion.

## 1 INTRODUCTION

Spontaneous abortion or miscarriage refers to clinically confirmed pregnancy loss before 12 weeks of gestation. The incidence of spontaneous abortion in clinically recognized pregnancies is 8‐20 percent and has increased rapidly in recent years.^1^ Recently, the impacts of the environment on the risk of spontaneous abortion have gained our attention due to industrial development and the deterioration of the ecological environment. Animal studies have shown that toxic metals accumulate in selected organs and pass through the placental barrier to the fetus, causing teratogenic and embryotoxic effects in a wide variety of species.^2^, ^3^ However, the evidence of metal toxicity in human embryos is deficient.^4^

Cadmium (Cd) is present in almost everything that we eat, drink, and breathe.^5^ The occupations with the highest potential for exposure include alloy production, battery production, pigment production and use, plastic production, and smelting and refining.^6^ For the general population, dietary cadmium comprises a predominant portion of cadmium exposure; the estimated dietary cadmium intake in European countries is 10‐30 µg per day,^7^ which increases with the consumption of certain foods, such as shellfish, offal, and rice. Another significant route of exposure is cigarette smoking, which is responsible for the assimilation of an estimated 0.2‐1.0 µg of cadmium with each cigarette smoked.^8^ In terms of clinical studies, a case‐control study of a small population of Chinese women found that women who experienced spontaneous abortion had higher hair cadmium levels than women with normal pregnancies.^9^

Another heavy metal, chromium (Cr), is also widespread in the environment, mainly in the form of chromium (III) and chromium (VI), which can be found in many consumer products, such as wood treated with copper dichromate, leather tanned with chromic sulfate, stainless steel cookware, and metal‐on‐metal hip replacements.^10^ Thus, while workers can suffer from extensive exposure to chromium during the production process,^11^ the predominant route of exposure for the general population is the ingestion of chromium in the diet. In an observational study, an increased risk of spontaneous abortion was observed in female workers with occupational chromium exposure compared to controls without occupational chromium exposure,^12^ but the general population was not affected.

Selenium (Se) is an essential nutrient for humans and is necessary for the function of enzymes involved in antioxidant defense, thyroid hormone metabolism, and redox control of intracellular reactions.^13^ Diet is also the major source of selenium. The recommended dietary allowance is 55 µg/d for adult females.^14^ It is believed that selenium deficiency in humans and animals may be the cause of some health complications. In the past few years, some authors have suggested that miscarriage is related to selenium deficiency^14^, ^15^; however, exposure to high levels of selenium can also have adverse effects. Workers in metal industries, paint manufacturing, and special trades may be exposed to higher levels of selenium, and short‐term or long‐term exposure to selenium can cause respiratory tract irritation, digestive difficulties, and even selenosis; furthermore, it is not known whether embryos are more susceptible to the effects of selenium, and selenium has been found in placental tissue, umbilical cord blood, fetal tissues, and breast milk.^16^

Additionally, arsenic (As) is common in the environment. Some occupations that may be exposed to arsenic include copper or lead smelting and wood treatment. Workers involved in the production or application of pesticides containing organic arsenicals may also be exposed to higher levels.^17^ In the general population, food is the largest source of arsenic.^18^ Although the toxicity of arsenic is widely recognized, studies of its embryotoxicity are controversial^19^, ^20^ and scarce, especially in general populations.

Mercury (Hg) is a naturally occurring metal that has several forms. These include metallic mercury, inorganic mercury compounds that are combinations with other elements, and organic mercury compounds combined with carbon; the most common of these compounds, methylmercury, is produced mainly by microscopic organisms in the water and soil and is built up in tissues of fish. The general population could be exposed to mercury by eating fish or shellfish contaminated with methylmercury, and occupational populations could breathe vapors in the air from dental work and industries that burn mercury‐containing fossil fuels. Currently, despite growing evidence of mercury‐associated embryotoxicity in a range of animal models, there is a paucity of data on the impact of mercury on humans.^21^

In conclusion, all of the toxic metals, including chromium, cadmium, arsenic, selenium, and mercury, are present everywhere in our lives and not just in particular work fields, and they have increasing relevance and concern to public health, especially reproductive health. However, the majority of existing studies have either concerned only populations with occupational exposure or have utilized ecologic exposure assessments; individual‐level evidence and lower‐level and chronic “real‐world” exposures to toxic metals that support the adverse effects of toxic metal exposure on pregnancy outcomes and embryotoxicity are still limited.

## 2 MATERIALS AND METHODS

### 2.1 Study population

We conducted a case‐control study of healthy pregnant women from January 2016 to October 2018 at Peking Union Medical College Hospital. For inclusion in the case group, healthy pregnant women needed to be clinically diagnosed with spontaneous abortion by criteria provided by ACR 2013, namely the patient needed to meet any one of the following four criteria: (a) crown‐rump length of equal to or greater than 7 mm without cardiac activity; (b) an mean sac diameter (MSD) equals to or greater than 25 mm without an embryo; (c) absence of embryo with a heartbeat 2 or more weeks after an ultrasound that showed a gestational sac without a yolk sac; and (d) absence of an embryo with a heartbeat at 11 days or more after an ultrasound that showed a gestational sac with a yolk sac.^22^ Exclusion criteria were as follows: occupational exposure to toxic metals; nonresident of Peking City; unavailable for follow‐up; continuous use of prescription drugs; diagnosis of high‐risk pregnancy, such as cesarean scar pregnancy; and pregnancy beyond 12 weeks of gestation. The inclusion criteria for the control group were women with an unwanted pregnancy, positive fetal cardiac activity on ultrasound at a maximum gestational age of 12 weeks, and a request for induced abortion. We compared the control group to the case group in terms of age; the number of cigarette smokers, consumers of alcohol, and coffee drinkers; and enrollment time. The ratio of cases to controls was 1:1. The exclusion criteria were the same as those for the case group. A total of 195 subjects participated in the study, and each of them had valid measurements of toxic metals.

All subjects were informed in detail about the design and aims of the research study. Those who agreed to participate read and signed a letter of informed consent. The research protocols were approved by the Ethics Committee of PUMCH and registered on Clinical Trials coding NCT03332706.

### 2.2 Data collection

#### 2.2.1 Assessment of covariates

We collected general information from the participants’ electronic medical records, and we obtained their reproductive histories via interviews. Information on other variables, such as diet, cigarette smoking, alcohol consumption, and possible occupational exposure to toxic metals, was also collected by questionnaire. Information on possible occupational exposure to toxic metals was collected via the following questions: Have you or your husband worked in stainless steel welding or at a leather tannery? Do you or your husband work in iron or steel foundries, or with or near wet cement, or in the electroplating, or wood preservation, or textile dyeing industries or in glass manufacturing?

Smokers were defined as those who had at least one cigarette per day, and alcohol consumers were defined as subjects who consumed more than 20 mL of liquor or beer per day. Coffee drinkers were defined as subjects who consumed one cup of coffee per day. All interviews and questionnaires were performed by specific trained personnel who analyzed the data.

#### 2.2.2 Toxic metal measurements

Blood and urine samples were collected from the subjects during each visit to the hospital. Prior to venipuncture, each subject's arm was washed with ultrapure water and disinfected with reagent‐grade alcohol. Three cubic centimeters of venous whole blood was collected with a butterfly catheter (19 gauge) into royal blue‐topped tubes containing EDTA (Vacutainer, B‐D 367734; Becton‐Dickinson), which was certified for a limited number of trace elements, including lead, for blood toxic metal analysis. In terms of urine, all patients were required to clean the vulva before collecting approximately 5 mL of clean urine from the middle part of the stream. All samples were stored in a freezer at −4°C and were analyzed using inductively coupled plasma mass spectrometry (ICP‐MS; Thermo Finnigan) within 7 days. For quality control, two quality control samples with clinically significant toxic metal levels were analyzed each time the instrument was set up for an analytical run, and the average of the two consecutive test results was used to determine whether the discrepancy was large enough to require a third analysis.

### 2.3 Statistics analysis

Differences in covariates between the case and control groups were assessed using Student's test for continuous variables such as age, gravidity, history of miscarriage, and blood and urine toxic metal levels; the chi‐squared test was used to compare categorical variables such as the number of smokers and number of alcohol drinkers. All of the items that were significantly different between the case and control groups were assessed by binary logistic regression analysis to calculate the odds ratios. All continuous variables are presented as averages (standard difference), and all categorical variables are presented as number (%). *P*‐values < 0.05 were considered indicative of statistically significant differences.

## 3 RESULTS

In our study, 195 women were enrolled, with 95 patients in the case group and 100 in the control group for a ratio of 1:1. Each contributed blood and urine specimens, and all of the samples were tested carefully. The baseline data for the two groups are shown in Table 1, and the groups were well matched in terms of age, number of cigarette smokers, consumers of alcoholic beverages, and coffee drinkers. Significant differences existed between gravidity, parity, and history of miscarriage (P1 = 0.013, P2 = 0.000, P3 = 0.000).

**Table 1:** Baseline of two groups

	Case group	Control group	*P* value
Age, y, average (standard difference)	34.28 (5.93)	34.47 (3.73)	0.790
Gravity*, average (standard difference)	2.36 (1.42)	2.84 (1.59)	0.027
Parity*, average (standard difference)	0.44 (0.61)	0.97 (0.78)	0.000
History of miscarriage* average (standard difference)	1.01 (1.33)	0.22 (0.66)	0.000
No. of smoker, n (%)	0	0	–
No. of consumer of alcohol beverage, n (%)	2 (2.1%)	3 (3%)	1.000
No. of coffee drinker, n (%)	8 (8.4%)	10 (10%)	0.807

* Indicates the items of significant differences.

The mean blood toxic metal levels were 1.12, 0.32, 117.76, 1.55, and 0.94 µg/L for chromium, cadmium, selenium, arsenic, and mercury, respectively, for the case group and 1.41, 0.22, 118.83, 1.32, and 0.84 µg/L for the control group (P5 = 0.157, P6 = 0.002*, P7 = 0.820, P8 = 0.182, P9 = 0.272), as described in Table 2.

**Table 2:** Blood level of toxic metals of two groups. (µg/L)

Toxic metals	Cr	Cd*	Se	As	Hg
Case group, average（standard difference）	1.12 (1.32)	0.32 (0.28)	117.76 (35.65)	1.55 (1.31)	0.94 (0.61)
Control group, n（%）	1.41 (1.47)	0.22 (0.11)	118.83 (29.82)	1.32 (1.05)	0.84 (0.58)
*P* value	0.157	0.002	0.820	0.182	0.272

* Indicates the items of significant differences.

In addition, the mean urine toxic metal levels for chromium, cadmium, selenium, arsenic, and mercury for the case group were 2.67, 0.46, 20.32, 28.77, and 0.49 µg/L, respectively, and 1.41, 0.22, 118.83, 1.32, and 0.84 µg/L for the control group (P4 = 0.046*, P5 = 696, P6 = 0.408, P7 = 0.763, P8 = 0.287). All the above data are shown in Table 3.

**Table 3:** Urine level of toxic metals of two groups. (µg/L)

Toxic metals	Cr*	Cd	Se	As	Hg
Case group, average（standard difference）	2.67 (3.94)	0.46 (0.49)	20.32 (13.22)	28.77 (34.71)	0.49 (0.41)
Control group, n（%）	1.71 (2.51)	0.49 (0.49)	22.00 (15.13)	27.31 (32.47)	0.64 (1.36)
*P* value	0.046	0.696	0.408	0.763	0.287

* Indicates the items of significant differences.

In the general population, the mean level of chromium in the blood is 2.5 µg/L,^23^, ^24^ and the geometric blood level of cadmium in the general population is 0.315 µg/L.^25^ We assumed that higher toxic metal levels would be associated with a greater possibility of miscarriage/spontaneous abortion; therefore, we used blood cadmium >0.4 µg/L and urine chromium >2 µg/L as our cutoff points and included them in a logistic recessive analysis with gravity <3, parity <2, and history of miscarriage >1. The odds ratios for spontaneous abortion in women who met the cutoffs for these biomarkers compared to those who did not were 1.26 (1.09, 1.85), 1.56 (1.23, 2.53), 1.39 (1.17, 1.98), 1.72 (1.21, 4.62), and 1.18 (1.06, 1.65), with P‐values of 0.003, 0.031, 0.003, 0.247, and 0.001, respectively, and all details are shown in Table 4.

**Table 4:** Logistic recessive analysis for possible biomarkers of embryotoxicity or spontaneous abortion

Toxic metals	Gravity ＜3*	Parity ＜2	History of miscarriage ＞2*	Blood level of Cd ＞0.4 µg/L*	Urine level of Cr ＞2 µg/L*
*P* value	0.003	0.247	0.001	0.003	0.031
OR( interval）	1.39 (1.17, 1.98)	1.72 (1.21, 4.62)	1.18 (1.06, 1.65)	1.26 (1.09, 1.85)	1.56 (1.23, 2.53)

* Indicates the items of significant differences.

## 4 DISCUSSION

In our study, we found two possible biomarkers for embryotoxicity, blood cadmium and urine chromium levels, among the toxic metals we tested in our subjects. Additionally, the odds ratio for spontaneous abortion with blood cadmium >0.4 µg/L, urine chromium >2 µg/L, gravidity <3, parity <2, and history of miscarriage >1 were 1.26 (1.09, 1.85), 1.56 (1.23, 2.53), 1.39 (1.17, 1.98), 1.72 (1.21, 4.62), and 1.18 (1.06, 1.65), with P‐values of 0.003, 0.031 0.003, 0.247, and 0.001, respectively.

Each of the two most common oxidation states, chromium (III) and chromium (VI), displays very different physical and biological properties; the chromium present in urine samples is mostly chromium (III),^26^ while whole‐blood chromium includes both types because Cr(VI) passes through cell membranes, while Cr(III) does not.^27^ Unlike occupational populations, the general population may be exposed to chromium through food, water, and air, in which chromium (III) is the main component. In our study, neither the case group nor the control group had occupational exposure to these metals, which also explains why the two groups differed in urine chromium levels but not in blood chromium levels. The difference in blood levels of chromium indicates its possible embryotoxicity, as Banu reported in a previous study.^28^

Usually, blood and urine cadmium levels are commonly used biomarkers of cadmium exposure; however, blood cadmium levels are more indicative of recent exposure than of the whole‐body burden, while urine cadmium levels mainly reflect the total body burden. Our study found a significant difference between the case and control groups in blood cadmium levels, but not in urine cadmium levels, which may also indicate that recent cadmium exposure is more likely to be related to embryotoxicity and spontaneous abortion rather than the whole‐body cadmium burden. Our conclusion regarding cadmium's embryotoxicity agrees with Thompson's findings in her review of the toxic effects on the reproductive system and the embryo.^29^

Regarding arsenic, once absorbed, arsenates are partially reduced to arsenites, yielding a mixture of As(III) and As(V) in the blood. Most inorganic arsenic is promptly excreted in the urine,^30^ which is consistent with our study findings that the urine arsenic level is much higher than the blood arsenic level.

Selenium metabolism involves several steps in which inorganic selenium is converted into selenoproteins or methylated metabolites of selenide and a small portion of selenium is eliminated primarily in the urine,^31^ which is also the reason why the blood selenium level is much higher than the urine selenium level.

There are limitations in our study. First, in our study, arsenic, mercury, and selenium levels were not significantly different between the case and control groups; however, we could not conclude that there is no possible association between these three elements and miscarriage or spontaneous abortion, and more studies about their embryotoxicity are needed. A second limitation lies in the case‐controlled study design; the weak sequential relationship between exposure and outcomes meant that the causal relationship between the biomarkers we chose and spontaneous abortion was not strong enough.
